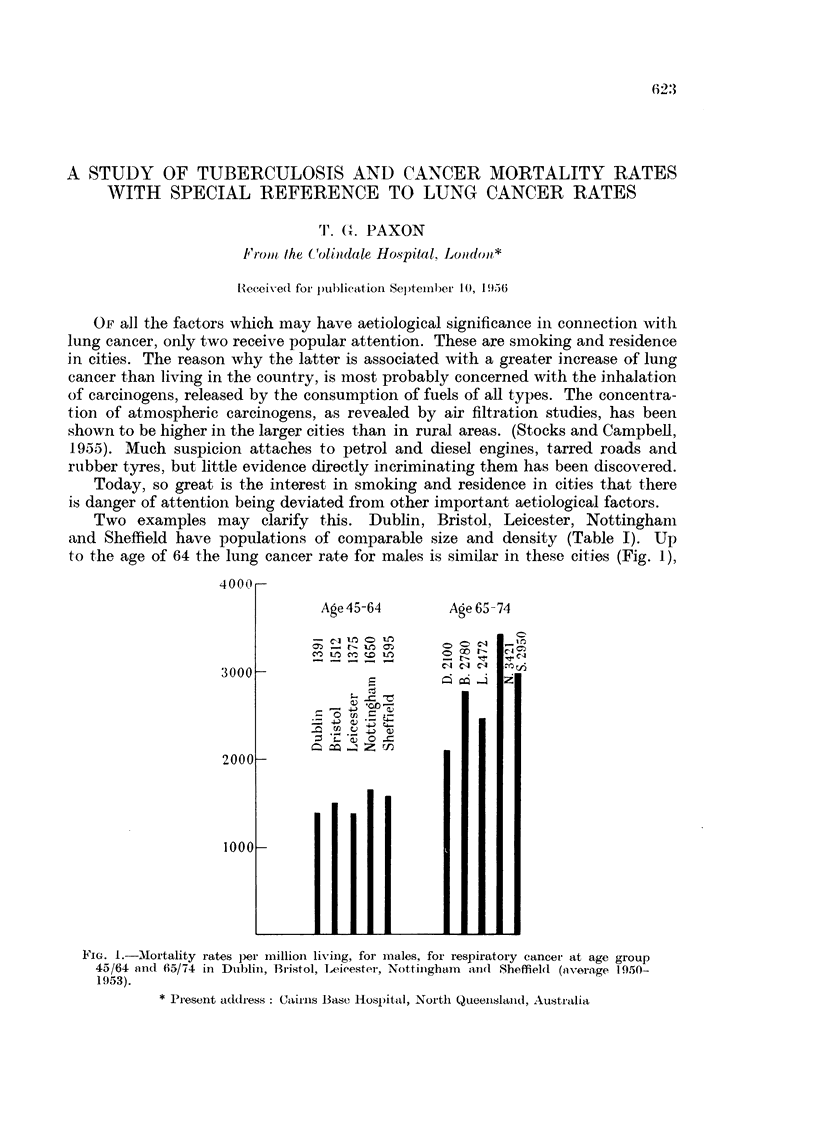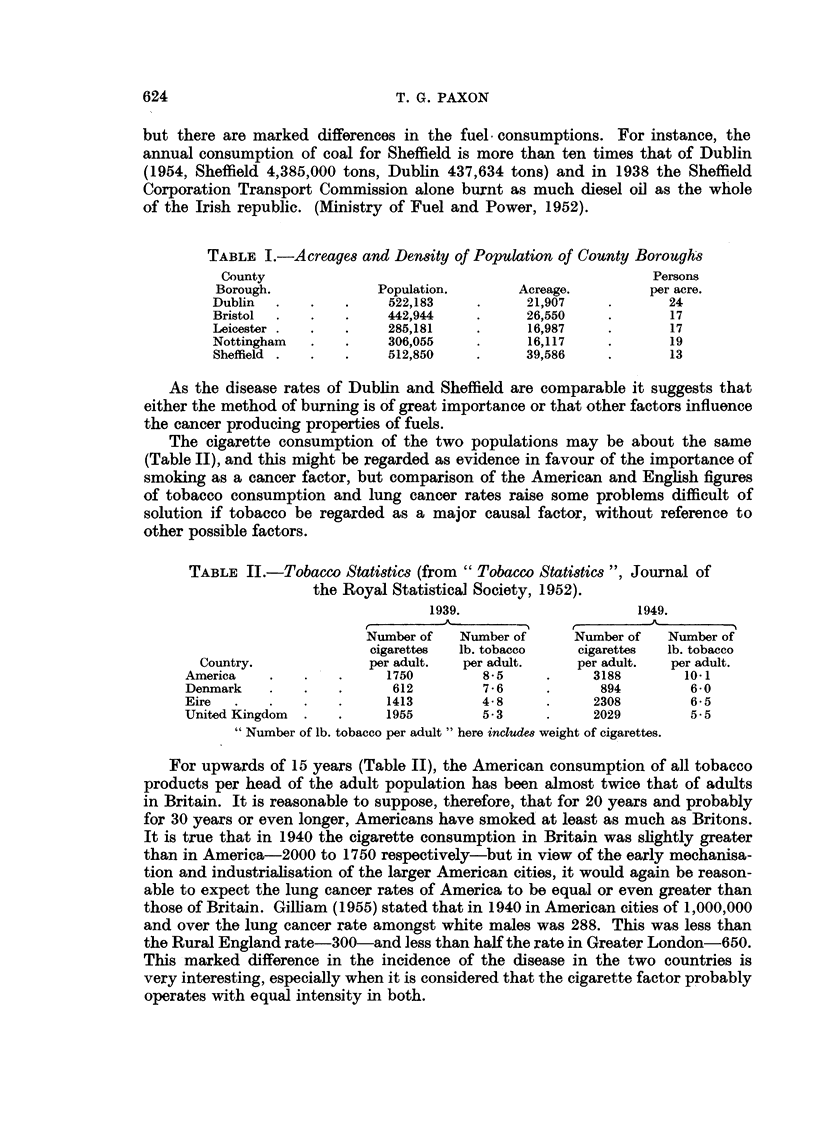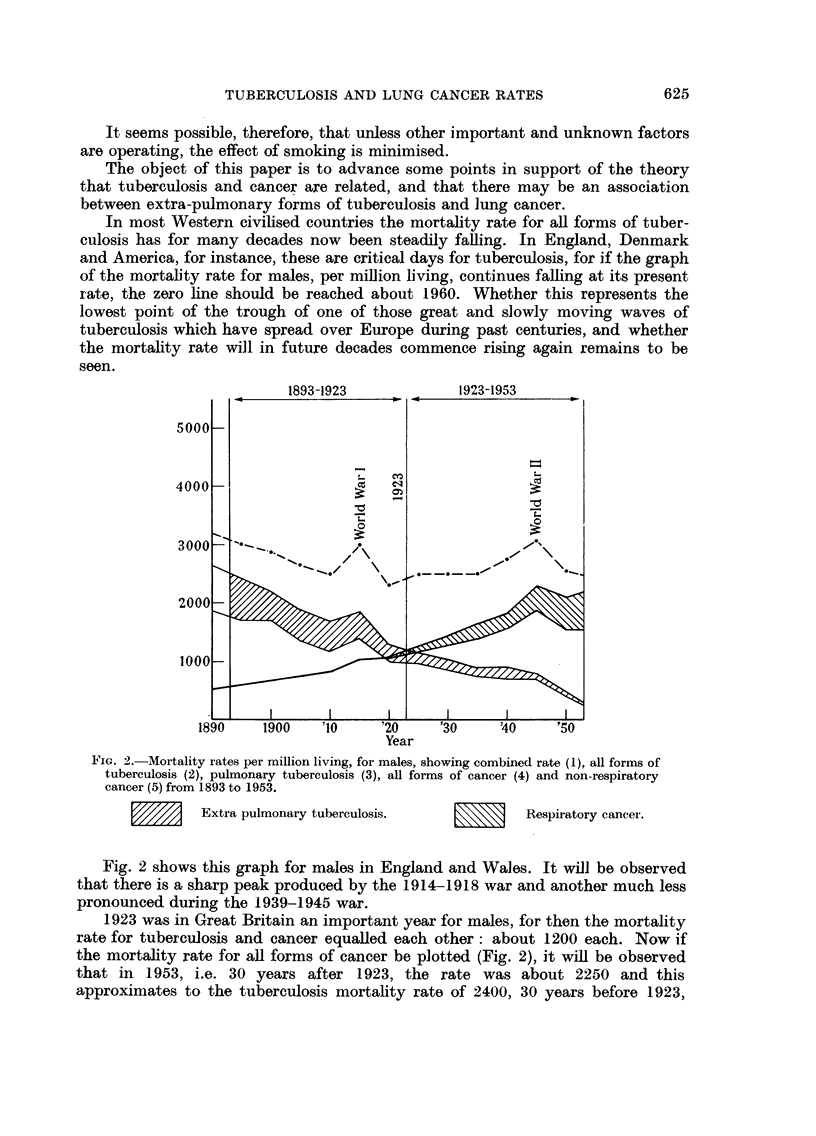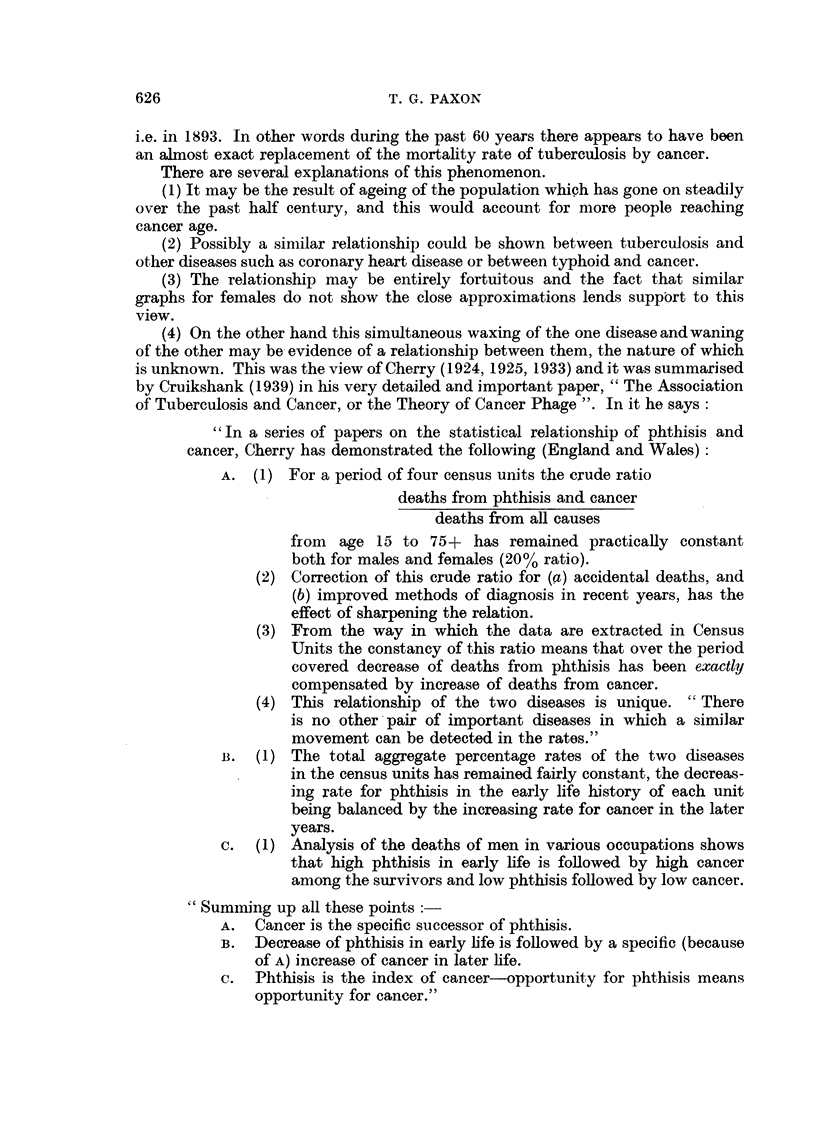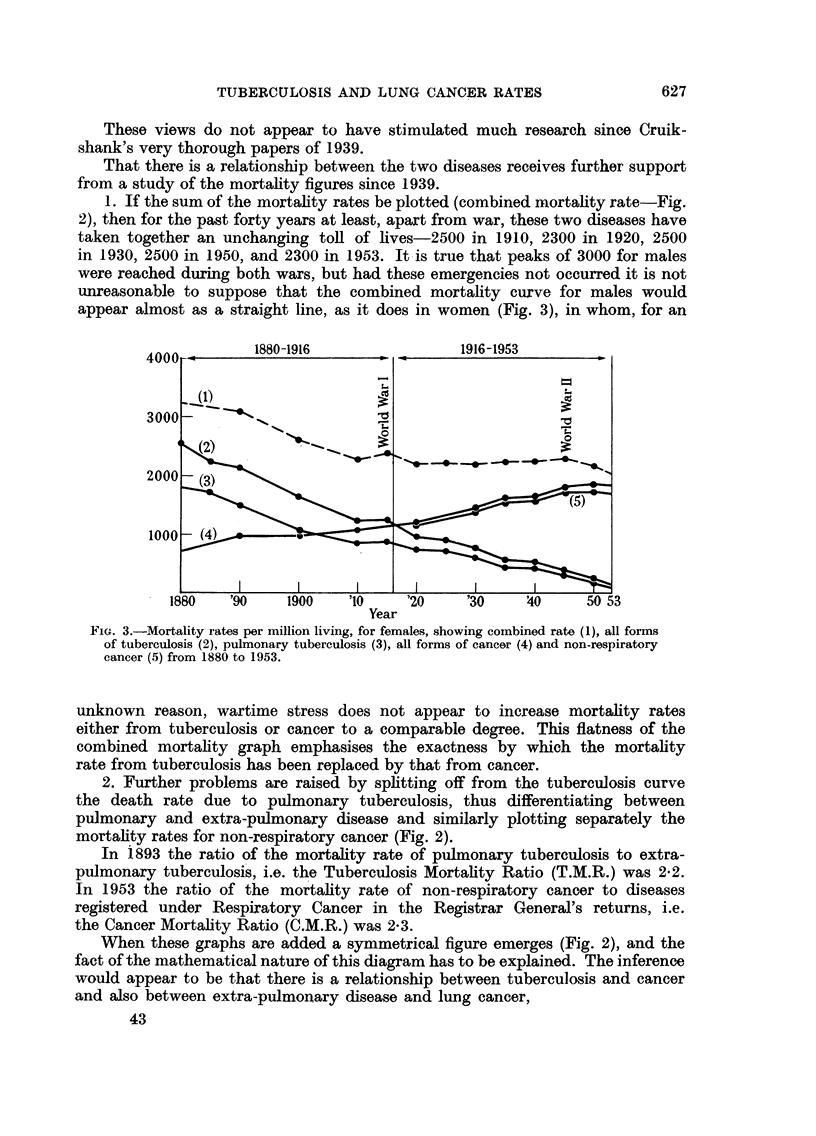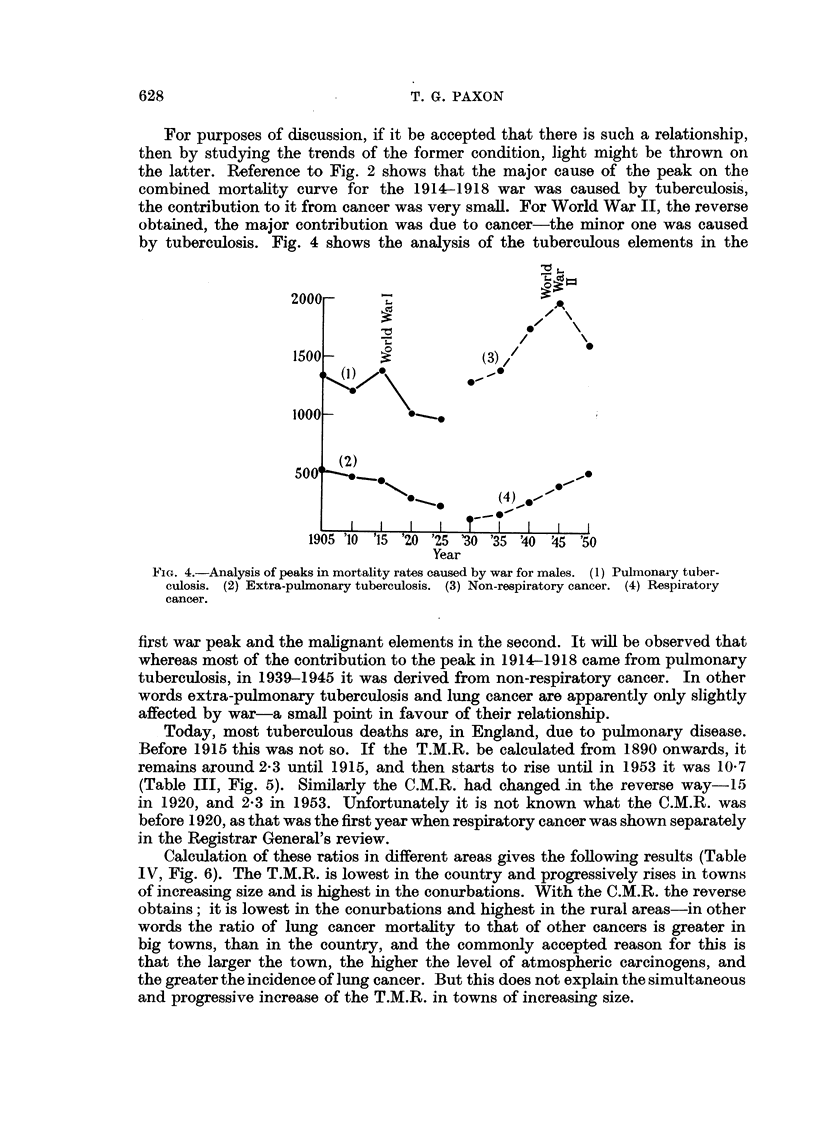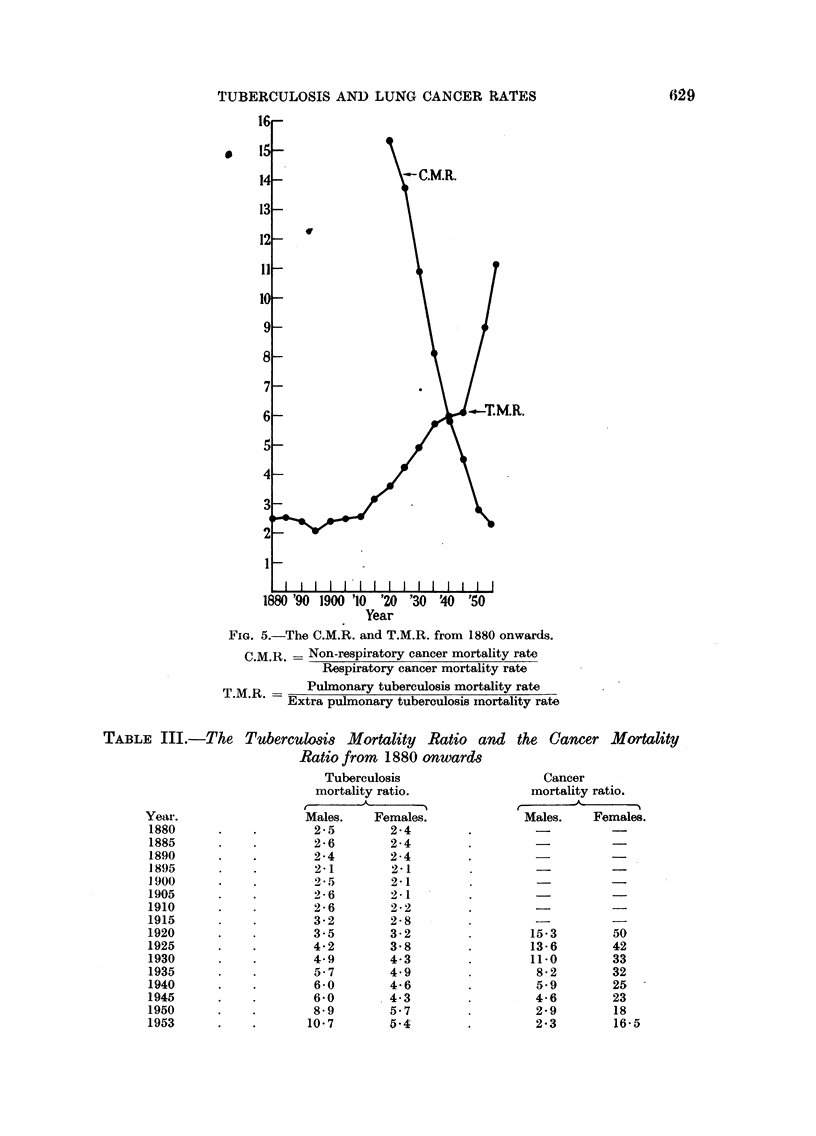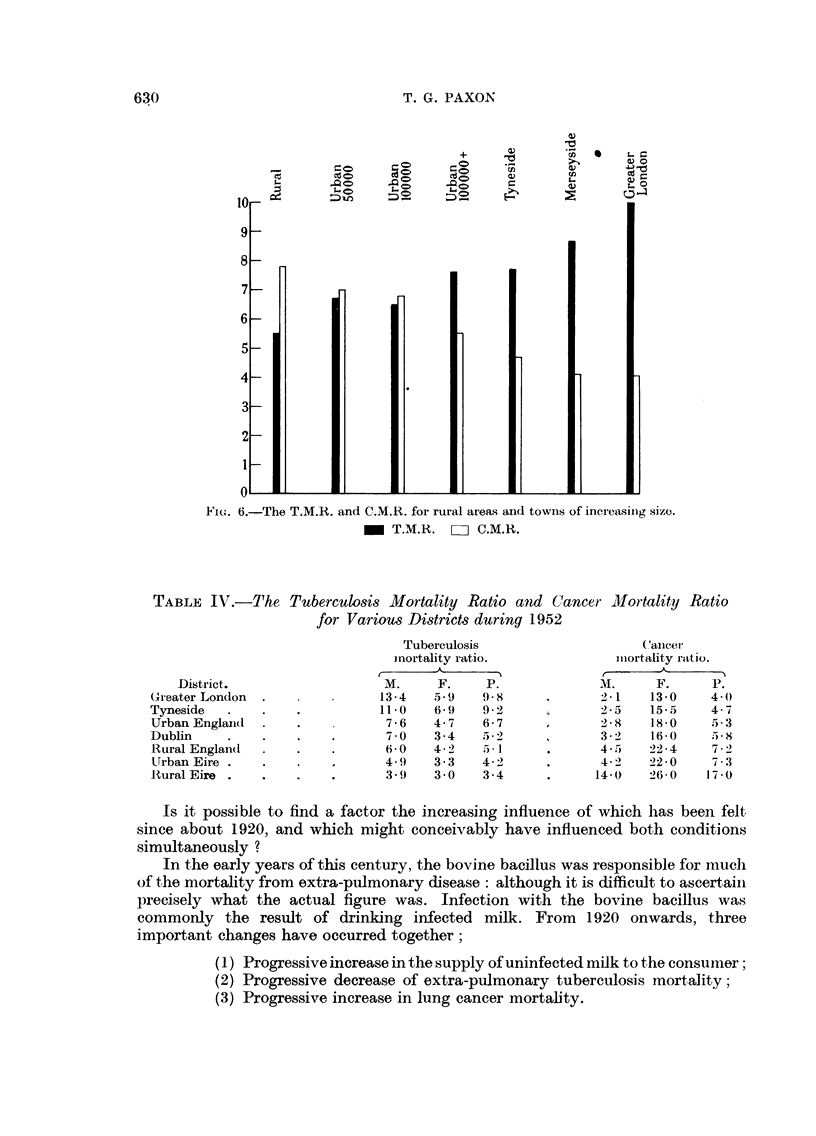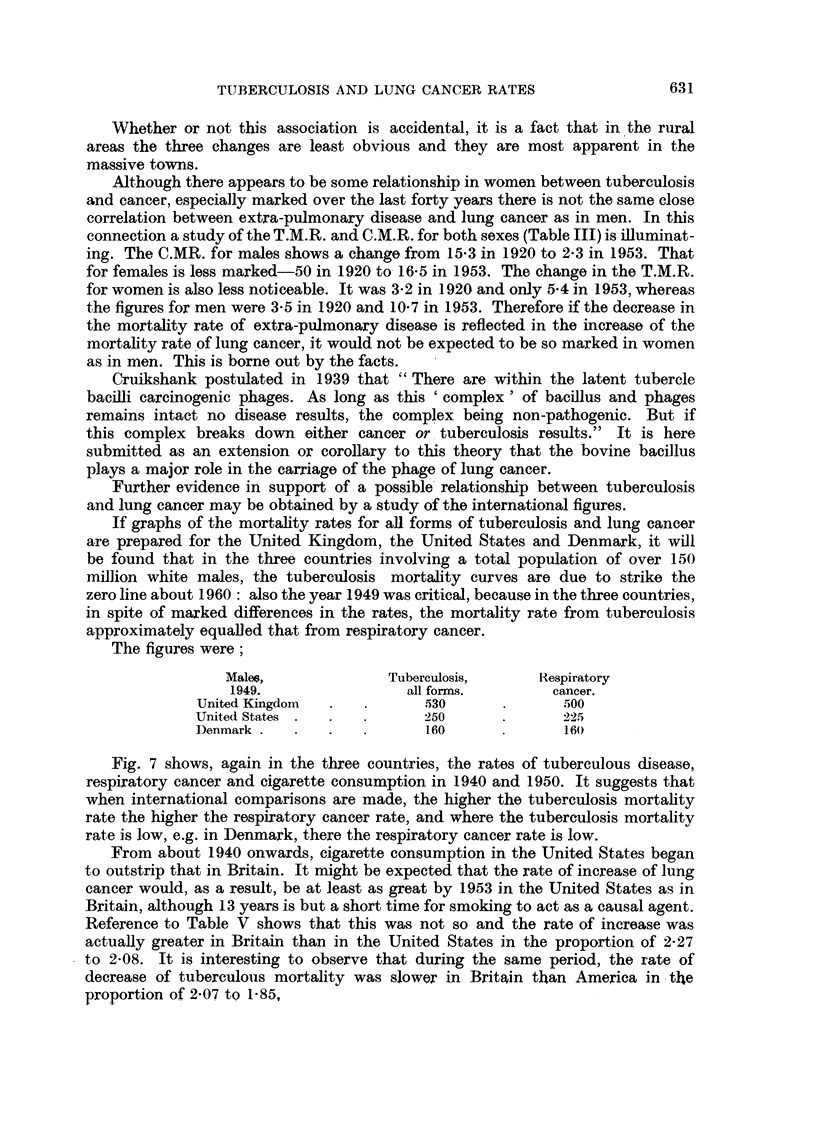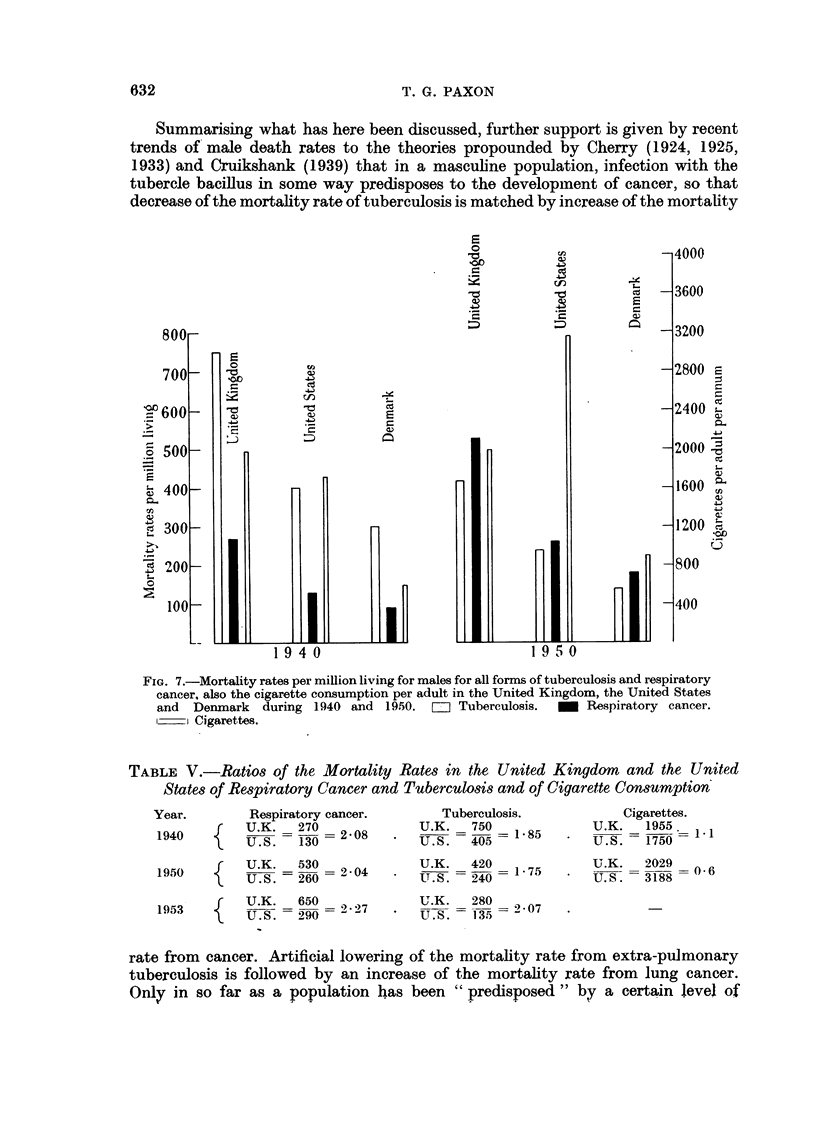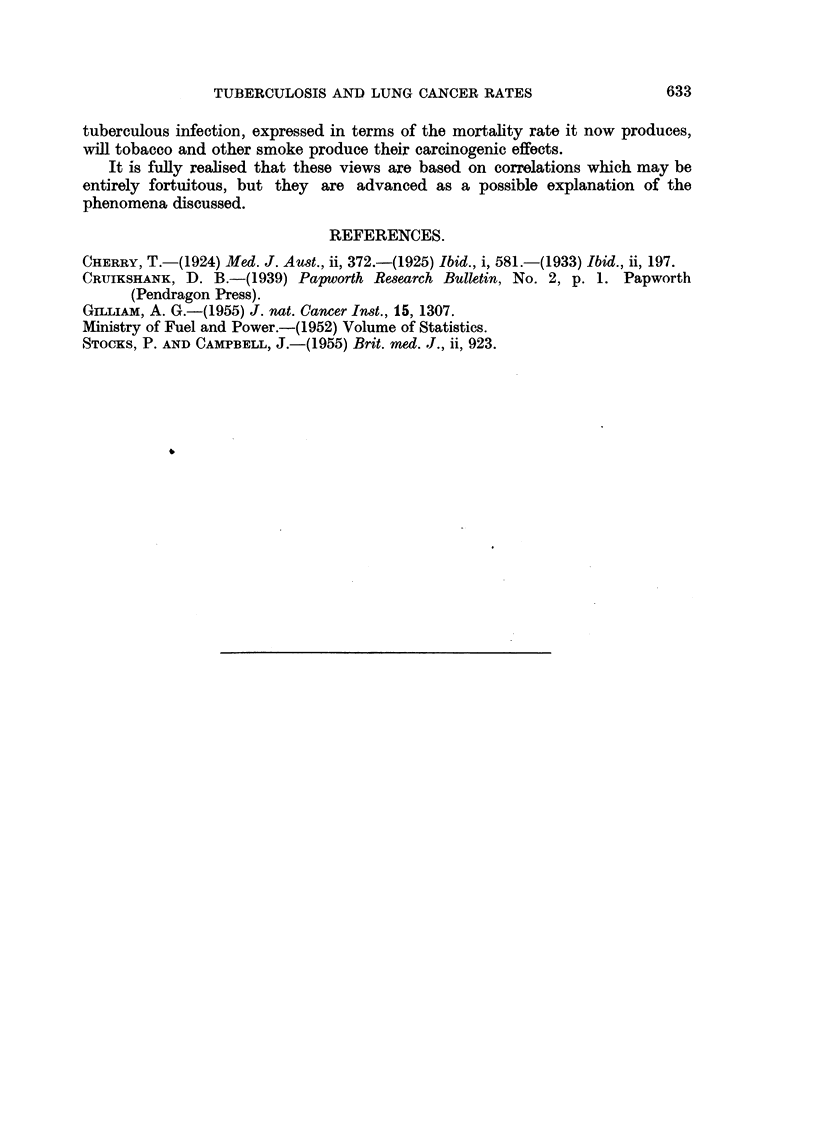# A Study of Tuberculosis and Cancer Mortality Rates with Special Reference to Lung Cancer Rates

**DOI:** 10.1038/bjc.1956.74

**Published:** 1956-12

**Authors:** T. G. Paxon


### A STUDY OF TUBERCULOSIS AND CANCER MORTALITY RATES WITH SPECIAL REFERENCE TO LUNG CANCER RATES

T. G. PAXON

*From the Colindale Hospital, London**

Received for publication September 10, 1956

Of all the factors which may have aetiological significance in connection with lung cancer, only two receive popular attention. These are smoking and residence in cities. The reason why the latter is associated with a greater increase of lung cancer than living in the country, is most probably concerned with the inhalation of carcinogens, released by the consumption of fuels of all types. The concentration of atmospheric carcinogens, as revealed by air filtration studies, has been shown to be higher in the larger cities than in rural areas. (Stocks and Campbell, 1955). Much suspicion attaches to petrol and diesel engines, tarred roads and rubber tyres, but little evidence directly incriminating them has been discovered.

Today, so great is the interest in smoking and residence in cities that there is danger of attention being deviated from other important aetiological factors.

Two examples may clarify this. Dublin, Bristol, Leicester, Nottingham and Sheffield have populations of comparable size and density (Table I). Up to the age of 64 the lung cancer rate for males is similar in these cities (Fig. 1),

Fig. 1.—Mortality rates per million living, for males, for respiratory cancer at age group 45/64 and 65/74 in Dublin, Bristol, Leicester, Nottingham and Sheffield (average 1950–1953).

* Present address : Cairns Base Hospital, North Queensland, Australia but there are marked differences in the fuel consumptions. For instance, the annual consumption of coal for Sheffield is more than ten times that of Dublin (1954, Sheffield 4,385,000 tons, Dublin 437,634 tons) and in 1938 the Sheffield Corporation Transport Commission alone burnt as much diesel oil as the whole of the Irish republic. (Ministry of Fuel and Power, 1952).

TABLE I.—*Acreages and Density of Population of County Boroughs*

| County Borough. | Population. | Acreage. | Persons per acre. |
|---|---|---|---|
| Dublin | 522,183 | 21,907 | 24 |
| Bristol | 442,944 | 26,550 | 17 |
| Leicester | 285,181 | 16,987 | 17 |
| Nottingham | 306,055 | 16,117 | 19 |
| Sheffield | 512,850 | 39,586 | 13 |

As the disease rates of Dublin and Sheffield are comparable it suggests that either the method of burning is of great importance or that other factors influence the cancer producing properties of fuels.

The cigarette consumption of the two populations may be about the same (Table II), and this might be regarded as evidence in favour of the importance of smoking as a cancer factor, but comparison of the American and English figures of tobacco consumption and lung cancer rates raise some problems difficult of solution if tobacco be regarded as a major causal factor, without reference to other possible factors.

TABLE II.—*Tobacco Statistics* (from "*Tobacco Statistics*", Journal of the Royal Statistical Society, 1952).

| | 1939. | | 1949. | |
|---|---|---|---|---|
| Country. | Number of cigarettes per adult. | Number of lb. tobacco per adult. | Number of cigarettes per adult. | Number of lb. tobacco per adult. |
| America | 1750 | 8·5 | 3188 | 10·1 |
| Denmark | 612 | 7·6 | 894 | 6·0 |
| Eire | 1413 | 4·8 | 2308 | 6·5 |
| United Kingdom | 1955 | 5·3 | 2029 | 5·5 |

"Number of lb. tobacco per adult" here *includes* weight of cigarettes.

For upwards of 15 years (Table II), the American consumption of all tobacco products per head of the adult population has been almost twice that of adults in Britain. It is reasonable to suppose, therefore, that for 20 years and probably for 30 years or even longer, Americans have smoked at least as much as Britons. It is true that in 1940 the cigarette consumption in Britain was slightly greater than in America—2000 to 1750 respectively—but in view of the early mechanisation and industrialisation of the larger American cities, it would again be reasonable to expect the lung cancer rates of America to be equal or even greater than those of Britain. Gilliam (1955) stated that in 1940 in American cities of 1,000,000 and over the lung cancer rate amongst white males was 288. This was less than the Rural England rate—300—and less than half the rate in Greater London—650. This marked difference in the incidence of the disease in the two countries is very interesting, especially when it is considered that the cigarette factor probably operates with equal intensity in both.

It seems possible, therefore, that unless other important and unknown factors are operating, the effect of smoking is minimised.

The object of this paper is to advance some points in support of the theory that tuberculosis and cancer are related, and that there may be an association between extra-pulmonary forms of tuberculosis and lung cancer.

In most Western civilised countries the mortality rate for all forms of tuberculosis has for many decades now been steadily falling. In England, Denmark and America, for instance, these are critical days for tuberculosis, for if the graph of the mortality rate for males, per million living, continues falling at its present rate, the zero line should be reached about 1960. Whether this represents the lowest point of the trough of one of those great and slowly moving waves of tuberculosis which have spread over Europe during past centuries, and whether the mortality rate will in future decades commence rising again remains to be seen.

FIG. 2.—Mortality rates per million living, for males, showing combined rate (1), all forms of tuberculosis (2), pulmonary tuberculosis (3), all forms of cancer (4) and non-respiratory cancer (5) from 1893 to 1953.

Extra pulmonary tuberculosis. Respiratory cancer.

Fig. 2 shows this graph for males in England and Wales. It will be observed that there is a sharp peak produced by the 1914–1918 war and another much less pronounced during the 1939–1945 war.

1923 was in Great Britain an important year for males, for then the mortality rate for tuberculosis and cancer equalled each other : about 1200 each. Now if the mortality rate for all forms of cancer be plotted (Fig. 2), it will be observed that in 1953, i.e. 30 years after 1923, the rate was about 2250 and this approximates to the tuberculosis mortality rate of 2400, 30 years before 1923, i.e. in 1893. In other words during the past 60 years there appears to have been an almost exact replacement of the mortality rate of tuberculosis by cancer.

There are several explanations of this phenomenon.

(1) It may be the result of ageing of the population which has gone on steadily over the past half century, and this would account for more people reaching cancer age.

(2) Possibly a similar relationship could be shown between tuberculosis and other diseases such as coronary heart disease or between typhoid and cancer.

(3) The relationship may be entirely fortuitous and the fact that similar graphs for females do not show the close approximations lends support to this view.

(4) On the other hand this simultaneous waxing of the one disease and waning of the other may be evidence of a relationship between them, the nature of which is unknown. This was the view of Cherry (1924, 1925, 1933) and it was summarised by Cruikshank (1939) in his very detailed and important paper, " The Association of Tuberculosis and Cancer, or the Theory of Cancer Phage ". In it he says :

> "In a series of papers on the statistical relationship of phthisis and cancer, Cherry has demonstrated the following (England and Wales) :
>
> A. (1) For a period of four census units the crude ratio
>
> $$\frac{\text{deaths from phthisis and cancer}}{\text{deaths from all causes}}$$
>
> from age 15 to 75+ has remained practically constant both for males and females (20% ratio).
>
> (2) Correction of this crude ratio for (*a*) accidental deaths, and (*b*) improved methods of diagnosis in recent years, has the effect of sharpening the relation.
>
> (3) From the way in which the data are extracted in Census Units the constancy of this ratio means that over the period covered decrease of deaths from phthisis has been *exactly* compensated by increase of deaths from cancer.
>
> (4) This relationship of the two diseases is unique. " There is no other pair of important diseases in which a similar movement can be detected in the rates."
>
> B. (1) The total aggregate percentage rates of the two diseases in the census units has remained fairly constant, the decreasing rate for phthisis in the early life history of each unit being balanced by the increasing rate for cancer in the later years.
>
> C. (1) Analysis of the deaths of men in various occupations shows that high phthisis in early life is followed by high cancer among the survivors and low phthisis followed by low cancer.
>
> " Summing up all these points :—
>
> A. Cancer is the specific successor of phthisis.
>
> B. Decrease of phthisis in early life is followed by a specific (because of A) increase of cancer in later life.
>
> C. Phthisis is the index of cancer—opportunity for phthisis means opportunity for cancer."

These views do not appear to have stimulated much research since Cruikshank's very thorough papers of 1939.

That there is a relationship between the two diseases receives further support from a study of the mortality figures since 1939.

1. If the sum of the mortality rates be plotted (combined mortality rate—Fig. 2), then for the past forty years at least, apart from war, these two diseases have taken together an unchanging toll of lives—2500 in 1910, 2300 in 1920, 2500 in 1930, 2500 in 1950, and 2300 in 1953. It is true that peaks of 3000 for males were reached during both wars, but had these emergencies not occurred it is not unreasonable to suppose that the combined mortality curve for males would appear almost as a straight line, as it does in women (Fig. 3), in whom, for an

FIG. 3.—Mortality rates per million living, for females, showing combined rate (1), all forms of tuberculosis (2), pulmonary tuberculosis (3), all forms of cancer (4) and non-respiratory cancer (5) from 1880 to 1953.

unknown reason, wartime stress does not appear to increase mortality rates either from tuberculosis or cancer to a comparable degree. This flatness of the combined mortality graph emphasises the exactness by which the mortality rate from tuberculosis has been replaced by that from cancer.

2. Further problems are raised by splitting off from the tuberculosis curve the death rate due to pulmonary tuberculosis, thus differentiating between pulmonary and extra-pulmonary disease and similarly plotting separately the mortality rates for non-respiratory cancer (Fig. 2).

In 1893 the ratio of the mortality rate of pulmonary tuberculosis to extra-pulmonary tuberculosis, i.e. the Tuberculosis Mortality Ratio (T.M.R.) was 2·2. In 1953 the ratio of the mortality rate of non-respiratory cancer to diseases registered under Respiratory Cancer in the Registrar General's returns, i.e. the Cancer Mortality Ratio (C.M.R.) was 2·3.

When these graphs are added a symmetrical figure emerges (Fig. 2), and the fact of the mathematical nature of this diagram has to be explained. The inference would appear to be that there is a relationship between tuberculosis and cancer and also between extra-pulmonary disease and lung cancer,

For purposes of discussion, if it be accepted that there is such a relationship, then by studying the trends of the former condition, light might be thrown on the latter. Reference to Fig. 2 shows that the major cause of the peak on the combined mortality curve for the 1914–1918 war was caused by tuberculosis, the contribution to it from cancer was very small. For World War II, the reverse obtained, the major contribution was due to cancer—the minor one was caused by tuberculosis. Fig. 4 shows the analysis of the tuberculous elements in the

Fig. 4.—Analysis of peaks in mortality rates caused by war for males. (1) Pulmonary tuberculosis. (2) Extra-pulmonary tuberculosis. (3) Non-respiratory cancer. (4) Respiratory cancer.

first war peak and the malignant elements in the second. It will be observed that whereas most of the contribution to the peak in 1914–1918 came from pulmonary tuberculosis, in 1939–1945 it was derived from non-respiratory cancer. In other words extra-pulmonary tuberculosis and lung cancer are apparently only slightly affected by war—a small point in favour of their relationship.

Today, most tuberculous deaths are, in England, due to pulmonary disease. Before 1915 this was not so. If the T.M.R. be calculated from 1890 onwards, it remains around 2·3 until 1915, and then starts to rise until in 1953 it was 10·7 (Table III, Fig. 5). Similarly the C.M.R. had changed in the reverse way—15 in 1920, and 2·3 in 1953. Unfortunately it is not known what the C.M.R. was before 1920, as that was the first year when respiratory cancer was shown separately in the Registrar General's review.

Calculation of these ratios in different areas gives the following results (Table IV, Fig. 6). The T.M.R. is lowest in the country and progressively rises in towns of increasing size and is highest in the conurbations. With the C.M.R. the reverse obtains ; it is lowest in the conurbations and highest in the rural areas—in other words the ratio of lung cancer mortality to that of other cancers is greater in big towns, than in the country, and the commonly accepted reason for this is that the larger the town, the higher the level of atmospheric carcinogens, and the greater the incidence of lung cancer. But this does not explain the simultaneous and progressive increase of the T.M.R. in towns of increasing size.

FIG. 5.—The C.M.R. and T.M.R. from 1880 onwards.

$$\text{C.M.R.} = \frac{\text{Non-respiratory cancer mortality rate}}{\text{Respiratory cancer mortality rate}}$$

$$\text{T.M.R.} = \frac{\text{Pulmonary tuberculosis mortality rate}}{\text{Extra pulmonary tuberculosis mortality rate}}$$

TABLE III.—*The Tuberculosis Mortality Ratio and the Cancer Mortality Ratio from* 1880 *onwards*

| | Tuberculosis mortality ratio. | | Cancer mortality ratio. | |
|---|---|---|---|---|
| Year. | Males. | Females. | Males. | Females. |
| 1880 | 2·5 | 2·4 | — | — |
| 1885 | 2·6 | 2·4 | — | — |
| 1890 | 2·4 | 2·4 | — | — |
| 1895 | 2·1 | 2·1 | — | — |
| 1900 | 2·5 | 2·1 | — | — |
| 1905 | 2·6 | 2·1 | — | — |
| 1910 | 2·6 | 2·2 | — | — |
| 1915 | 3·2 | 2·8 | — | — |
| 1920 | 3·5 | 3·2 | 15·3 | 50 |
| 1925 | 4·2 | 3·8 | 13·6 | 42 |
| 1930 | 4·9 | 4·3 | 11·0 | 33 |
| 1935 | 5·7 | 4·9 | 8·2 | 32 |
| 1940 | 6·0 | 4·6 | 5·9 | 25 |
| 1945 | 6·0 | 4·3 | 4·6 | 23 |
| 1950 | 8·9 | 5·7 | 2·9 | 18 |
| 1953 | 10·7 | 5·4 | 2·3 | 16·5 |

FIG. 6.—The T.M.R. and C.M.R. for rural areas and towns of increasing size.
■ T.M.R. □ C.M.R.

TABLE IV.—*The Tuberculosis Mortality Ratio and Cancer Mortality Ratio for Various Districts during* 1952

| District. | Tuberculosis mortality ratio. M. | F. | P. | Cancer mortality ratio. M. | F. | P. |
|---|---|---|---|---|---|---|
| Greater London | 13·4 | 5·9 | 9·8 | 2·1 | 13·0 | 4·0 |
| Tyneside | 11·0 | 6·9 | 9·2 | 2·5 | 15·5 | 4·7 |
| Urban England | 7·6 | 4·7 | 6·7 | 2·8 | 18·0 | 5·3 |
| Dublin | 7·0 | 3·4 | 5·2 | 3·2 | 16·0 | 5·8 |
| Rural England | 6·0 | 4·2 | 5·1 | 4·5 | 22·4 | 7·2 |
| Urban Eire | 4·9 | 3·3 | 4·2 | 4·2 | 22·0 | 7·3 |
| Rural Eire | 3·9 | 3·0 | 3·4 | 14·0 | 26·0 | 17·0 |

Is it possible to find a factor the increasing influence of which has been felt since about 1920, and which might conceivably have influenced both conditions simultaneously ?

In the early years of this century, the bovine bacillus was responsible for much of the mortality from extra-pulmonary disease : although it is difficult to ascertain precisely what the actual figure was. Infection with the bovine bacillus was commonly the result of drinking infected milk. From 1920 onwards, three important changes have occurred together ;

(1) Progressive increase in the supply of uninfected milk to the consumer ;
(2) Progressive decrease of extra-pulmonary tuberculosis mortality ;
(3) Progressive increase in lung cancer mortality.

Whether or not this association is accidental, it is a fact that in the rural areas the three changes are least obvious and they are most apparent in the massive towns.

Although there appears to be some relationship in women between tuberculosis and cancer, especially marked over the last forty years there is not the same close correlation between extra-pulmonary disease and lung cancer as in men. In this connection a study of the T.M.R. and C.M.R. for both sexes (Table III) is illuminating. The C.MR. for males shows a change from 15·3 in 1920 to 2·3 in 1953. That for females is less marked—50 in 1920 to 16·5 in 1953. The change in the T.M.R. for women is also less noticeable. It was 3·2 in 1920 and only 5·4 in 1953, whereas the figures for men were 3·5 in 1920 and 10·7 in 1953. Therefore if the decrease in the mortality rate of extra-pulmonary disease is reflected in the increase of the mortality rate of lung cancer, it would not be expected to be so marked in women as in men. This is borne out by the facts.

Cruikshank postulated in 1939 that " There are within the latent tubercle bacilli carcinogenic phages. As long as this ' complex ' of bacillus and phages remains intact no disease results, the complex being non-pathogenic. But if this complex breaks down either cancer *or* tuberculosis results." It is here submitted as an extension or corollary to this theory that the bovine bacillus plays a major role in the carriage of the phage of lung cancer.

Further evidence in support of a possible relationship between tuberculosis and lung cancer may be obtained by a study of the international figures.

If graphs of the mortality rates for all forms of tuberculosis and lung cancer are prepared for the United Kingdom, the United States and Denmark, it will be found that in the three countries involving a total population of over 150 million white males, the tuberculosis mortality curves are due to strike the zero line about 1960 : also the year 1949 was critical, because in the three countries, in spite of marked differences in the rates, the mortality rate from tuberculosis approximately equalled that from respiratory cancer.

The figures were ;

| Males, 1949. | Tuberculosis, all forms. | Respiratory cancer. |
|---|---|---|
| United Kingdom | 530 | 500 |
| United States | 250 | 225 |
| Denmark | 160 | 160 |

Fig. 7 shows, again in the three countries, the rates of tuberculous disease, respiratory cancer and cigarette consumption in 1940 and 1950. It suggests that when international comparisons are made, the higher the tuberculosis mortality rate the higher the respiratory cancer rate, and where the tuberculosis mortality rate is low, e.g. in Denmark, there the respiratory cancer rate is low.

From about 1940 onwards, cigarette consumption in the United States began to outstrip that in Britain. It might be expected that the rate of increase of lung cancer would, as a result, be at least as great by 1953 in the United States as in Britain, although 13 years is but a short time for smoking to act as a causal agent. Reference to Table V shows that this was not so and the rate of increase was actually greater in Britain than in the United States in the proportion of 2·27 to 2·08. It is interesting to observe that during the same period, the rate of decrease of tuberculous mortality was slower in Britain than America in the proportion of 2·07 to 1·85.

Summarising what has here been discussed, further support is given by recent trends of male death rates to the theories propounded by Cherry (1924, 1925, 1933) and Cruikshank (1939) that in a masculine population, infection with the tubercle bacillus in some way predisposes to the development of cancer, so that decrease of the mortality rate of tuberculosis is matched by increase of the mortality

Fig. 7.—Mortality rates per million living for males for all forms of tuberculosis and respiratory cancer, also the cigarette consumption per adult in the United Kingdom, the United States and Denmark during 1940 and 1950. ▭ Tuberculosis. ■ Respiratory cancer. ▭ Cigarettes.

Table V.—*Ratios of the Mortality Rates in the United Kingdom and the United States of Respiratory Cancer and Tuberculosis and of Cigarette Consumption*

| Year. | Respiratory cancer. | Tuberculosis. | Cigarettes. |
|---|---|---|---|
| 1940 | $\frac{U.K.}{U.S.} = \frac{270}{130} = 2\cdot08$ | $\frac{U.K.}{U.S.} = \frac{750}{405} = 1\cdot85$ | $\frac{U.K.}{U.S.} = \frac{1955}{1750} = 1\cdot1$ |
| 1950 | $\frac{U.K.}{U.S.} = \frac{530}{260} = 2\cdot04$ | $\frac{U.K.}{U.S.} = \frac{420}{240} = 1\cdot75$ | $\frac{U.K.}{U.S.} = \frac{2029}{3188} = 0\cdot6$ |
| 1953 | $\frac{U.K.}{U.S.} = \frac{650}{290} = 2\cdot27$ | $\frac{U.K.}{U.S.} = \frac{280}{135} = 2\cdot07$ | — |

rate from cancer. Artificial lowering of the mortality rate from extra-pulmonary tuberculosis is followed by an increase of the mortality rate from lung cancer. Only in so far as a population has been "predisposed" by a certain level of tuberculous infection, expressed in terms of the mortality rate it now produces, will tobacco and other smoke produce their carcinogenic effects.

It is fully realised that these views are based on correlations which may be entirely fortuitous, but they are advanced as a possible explanation of the phenomena discussed.

### REFERENCES.

CHERRY, T.—(1924) *Med. J. Aust.*, ii, 372.—(1925) *Ibid.*, i, 581.—(1933) *Ibid.*, ii, 197.

CRUIKSHANK, D. B.—(1939) *Papworth Research Bulletin*, No. 2, p. 1. Papworth (Pendragon Press).

GILLIAM, A. G.—(1955) *J. nat. Cancer Inst.*, **15**, 1307.

Ministry of Fuel and Power.—(1952) Volume of Statistics.

STOCKS, P. AND CAMPBELL, J.—(1955) *Brit. med. J.*, ii, 923.